# Antihypertensive Effect of Dietary β-Conglycinin in the Spontaneously Hypertensive Rat (SHR)

**DOI:** 10.3390/metabo12050422

**Published:** 2022-05-08

**Authors:** Koji Kawabeta, Masahiro Yuasa, Michihiro Sugano, Kazunori Koba

**Affiliations:** 1 Graduate School of Human Health Science, University of Nagasaki, Siebold, Nagasaki 851-2915, Japan; kawabeta@sun.ac.jp; 2 Department of Health and Nutrition, Faculty of Health Management, Nagasaki International University, 2825715 Huis Ten Bosch, Nagasaki 859-3298, Japan; 3 Graduate School of Human Development and Environment, Kobe University, 3-11 Tsurukabuto, Nada-ku, Kobe 657-8501, Japan; yuasa@people.kobe-u.ac.jp; 4 Faculty of Nursing and Nutrition, University of Nagasaki, Siebold, Nagasaki 851-2195, Japan; 5 School of Agriculture, Kyushu University, Fukuoka 813-0043, Japan; suganomi@deluxe.ocn.ne.jp; 6 Faculty of Environmental and Symbiotic Sciences, Prefectural University of Kumamoto, Kumamoto 862-8502, Japan

**Keywords:** β-conglycinin, spontaneously hypertensive rat, adiponectin, nitric oxide, renin–angiotensin system

## Abstract

Dietary β-conglycinin has been shown to increase plasma adiponectin concentration and decrease visceral adipose tissue weight in rats. Since adiponectin is one of the factors regulating blood pressure, as well as modulating lipid metabolism, we examined whether dietary β-conglycinin affects blood pressure in spontaneously hypertensive rats. The experimental diets were prepared according to the AIN-93G formula containing 20% protein, either casein (Control) or casein replaced with soy protein isolate (SOY) or β-conglycinin (β-CON) at the proportion of 50%. Male rats (SHR/Izm, 6 wk-old) were fed the diets for 7 weeks. The SOY compared with the Control significantly suppressed the blood pressure both at week 4 (*p* = 0.011, Control vs. SOY) and thereafter, and β-CON had even higher suppression (*p* = 0.0002, Control vs. β-CON). SOY and β-CON increased plasma adiponectin concentration followed by an increase in plasma nitric oxide and possibly a decreasing trend of gene expressions of angiotensinogen in the liver and renin in the kidney. The results indicated suppression by β-conglycinin of increasing blood pressure through an enhancement of plasma adiponectin, probably in combination with a regulation of the renin–angiotensin system in spontaneously hypertensive rats.

## 1. Introduction

Hypertension is an important component of metabolic syndrome and is considered one of the major risk factors that promotes arteriosclerosis, along with obesity, dyslipidemia, and hyperglycemia [1,2]. These metabolic syndrome component factors can largely be influenced by lifestyle modifications, such as changes in dietary patterns and physical activities [3]. It has been suggested that several dietary components modulate blood pressure [4,5], and dietary protein is one of the candidates [6].

Dietary soy protein has been shown to modulate lipid metabolism in rodents [7,8]. Beta-conglycinin is one of the exclusive components of soy protein, composing approximately 23% of soy protein [9]. Previous studies demonstrated that dietary β-conglycinin lowered visceral adipose tissue weight and liver triglyceride concentration in rodents to a greater extent compared with soy protein [8,10,11]. The effect could be at least partly due to a decrease in fatty acid synthesis and an increase in fatty acid β-oxidation in the liver [10,12]. Dietary β-conglycinin also increased plasma adiponectin concentration in rats [11]. Adiponectin, one of the adipocytokines mainly secreted from adipocytes [13], has been suggested not only to modulate lipid metabolism but also to regulate blood pressure [14]. Moreover, adiponectin could stimulate the endothelium production of nitric oxide (NO) [14], an important mediator of endothelial function such as vasodilation and angiogenesis [15,16]. The available evidence suggests that dietary β-conglycinin could modulate blood pressure by increasing serum adiponectin concentration.

Blood pressure is known to be regulated by various factors other than adiponectin, such as the renin–angiotensin system [17], prostaglandins [18], and renal sodium metabolism [18]. Prostaglandins, such as prostaglandin E_2_ and prostacyclin, are vasodilative [19]. They are produced as needed from arachidonic acid in membrane phospholipids to exert an antihypertensive effect. For renal sodium metabolism, the insufficient excretion of salt and water due to impaired kidney function can cause an increase in blood volume and then exert an increase in blood pressure [18]. The renin–angiotensin system has been recognized as one of the important factors in the physiological regulation of blood pressure [17]. In addition, the renin–angiotensin system plays a pathophysiological role in the development of cardiovascular disease with hypertension [19]. Although there is an in vitro study suggesting that β-conglycinin suppressed angiotensin-converting enzyme (ACE) activity [20], it is unclear whether successive dietary ingestion of β-conglycinin can regulate blood pressure.

The present study, therefore, investigated how dietary β-conglycinin modulates blood pressure through the adiponectin and renin–angiotensin systems as well as lipid metabolism in the spontaneously hypertensive rat (SHR).

## 2. Results

### 2.1. Growth Parameters

In the present study, male Sprague Dawley rats were fed purified diets containing 20% protein, either casein (Control), casein replaced with soy protein isolate (SOY), or casein replaced with β-conglycinin (β-CON) at the proportion of 50%, for 48 days. As a result, no significant difference was observed in food intake, body weight gain, or food efficiency among the groups (Table 1). The results indicated that the amounts of individual nutrients consumed other than the protein source were comparable among the groups.

Liver and white adipose tissue weights were also comparable among the groups. The interscapular brown adipose tissue weight was heavy in the order of the β-CON > SOY > Control groups, and the difference between the β-CON and Control groups was statistically significant (*p* = 0.043).

### 2.2. Measurement of Blood Pressure

**Figure 1:**
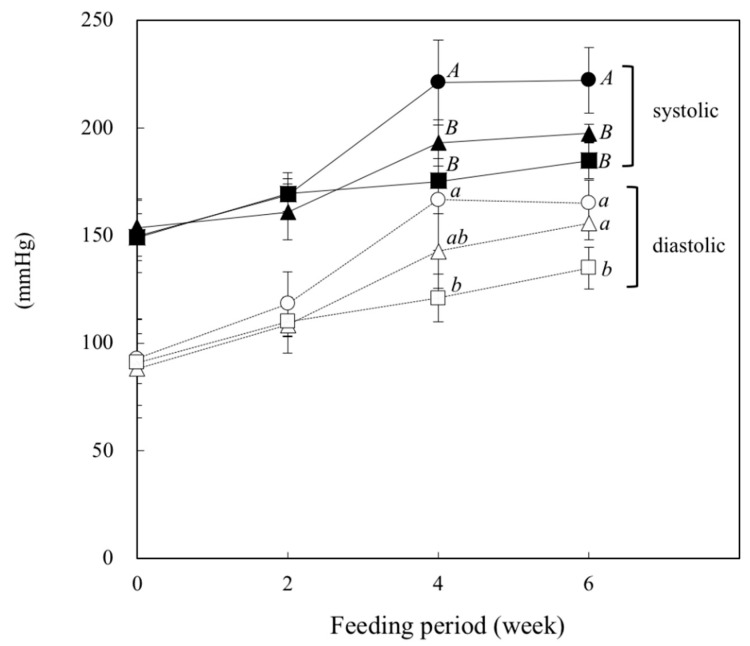
Effect of dietary β-conglycinin on systolic and diastolic blood pressures in SHR. During the feeding period, systolic and diastolic blood pressures were measured every 2 weeks by a tail-cuff method. ● and ○, Control (20% casein diet); ▲and △, SOY (the diet containing 10% soy protein isolate with 10% casein); ■ and □, β-CON (the diet containing 10% β-conglycinin with 10% casein). Values are expressed as mean ± SD of six rats. *^A^*^,*B*^ Values without sharing a common letter are significantly different at *p* < 0.05.

As shown in Figure 1, the systolic blood pressure (SBP) and diastolic blood pressure (DBP) measured by the tail-cuff method increased gradually with the feeding period in all groups of rats. At week 4, the SBP in the β-CON and SOY groups was significantly lower than that in the Control group (*p* = 0.0002, Control vs. β-CON.; *p* = 0.011, Control vs. SOY). The protein-dependent difference was also observed at week 6. The DBP at week 4 and thereafter was also significantly lower in the β-CON group than in the Control group (*p* = 0.001 at week 4, Control vs. β-CON). The DBP in the SOY group was between that in the Control and β-CON groups at weeks 4 and 6.

### 2.3. Plasma Biochemical Analysis

Concentrations of plasma triglyceride and phospholipid were comparable among the groups (Table 2). Cholesterol concentration in the SOY group was significantly lower (*p* = 0.014), whereas that of the β-CON group tended to be lower than that in the Control group. Free fatty acid (FFA) concentration in the β-CON group was significantly lower than that in the Control group (*p* = 0.047). Plasma concentrations of glucose and insulin were comparable among the groups. Plasma adiponectin level was high in the order of β-CON > SOY > Control groups, and the difference between the Control and β-CON groups was statistically significant (*p* = 0.027). The leptin level was comparable among the groups. The nitric oxide (NO) metabolite (NOx; NO_2_^−^+NO_3_^−^) level was 29% higher in the β-CON group, whereas it was 11% higher in the SOY group than in the Control group, though the difference was not statistically significant (*p =* 0.056, Control vs. β-CON). ACE activity was comparable among the groups.

### 2.4. Liver Lipids

The triglyceride concentration in the liver was significantly lower in the SOY group than in the Control group (*p* = 0.043) (Table 3). A similar trend was also observed between the β-CON and Control groups. The concentrations of cholesterol and phospholipid were comparable among the groups.

### 2.5. Enzyme Activities in the Liver

Liver cytosolic fatty acid synthase (FAS) activity in the SOY group was significantly lower (*p* = 0.006), whereas it tended to be lower in the β-CON group than that in the Control group (*p* = 0.065) (Table 4). A similar trend was observed in cytosolic glucose-6-phosphate dehydrogenase (G6PDH) activity. The activities of cytosolic malic enzyme and microsomal phosphatidate phosphohydrolase (PAP) were comparable among the groups. The activity of mitochondrial carnitine-palmitoyltransferase (CPT) tended to be higher in the SOY and β-CON groups than in the Control group, though the difference was not statistically significant.

### 2.6. Gene Expressions in the Tissues

**Figure 2:**
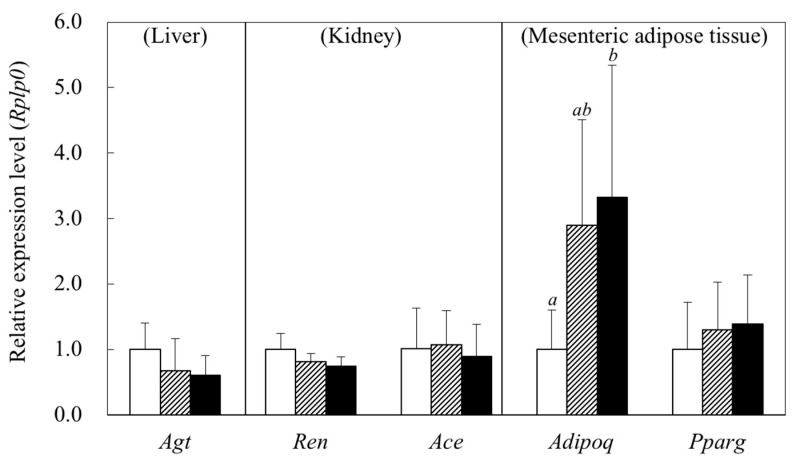
Effect of dietary β-conglycinin on gene expressions in liver, kidney, and mesenteric adipose tissue in SHR. *Agt*, angiotensinogen; *Ren*, renin; *Ace*, angiotensin converting enzyme; *Adipoq*, adiponectin; *Pparg*, peroxisome proliferator-activated receptor ganma; *Rplp0*, acidic ribosomal phosphoprotein. The mRNA levels were normalized by *Rplp0* expression and expressed as the fold induction relative to the Control. The gene expressions were calculated using the 2^−ΔΔC^*_T_* method. Open bar, Control (20% casein diet); hatched bar, SOY (the diet containing 10% soy protein isolate with 10% casein); solid bar, β-CON (the diet containing 10% β-conglycinin with 10% casein). Rats were given free access to the diets for 48 days. Values are expressed as mean ± SD of six rats. *^a^*^,*b*^ Values without sharing a common letter are significantly different at *p* < 0.05.

Dietary protein did not significantly affect the levels of mRNA encoding angiotensinogen (*Agt*) in the liver and renin (*Ren*) in the kidney (Control vs. β-CON.; *p* = 0.316 for *Agt* and *p* = 0.220 for *Ren*, respectively) (Figure 2). The level of mRNA encoding ACE (*Ace*) was comparable among the groups.

In the mesenteric adipose tissue, the level of mRNA encoding adiponectin (*Adipoq*) was significantly higher in the β-CON group than in the Control group (*p* = 0.048). A similar but less clear trend was also observed in the level of mRNA encoding proliferator-activated receptor *γ* (*Pparg*).

## 3. Discussion

In the present study, we examined how dietary β-conglycinin regulates blood pressure in SHR, a useful animal model of spontaneous hypertension. Feeding of β-conglycinin compared with casein (Control) significantly suppressed the elevation of SBP and DBP in SHR, indicating an antihypertensive effect of β-conglycinin. This result was consistent with the observation that β-conglycinin lowered SBP in the streptozotocin-induced diabetic nephropathy rats [21]. Moreover, the peptide derived from soy protein was reported to suppress the SBP in SHR [22]. Therefore, it is likely that the β-conglycinin-dependent antihypertensive effect could be attributable to its specific peptide fraction.

In the present study, the plasma adiponectin concentration was significantly higher in rats fed the β-CON diet than in those fed the Control diet, consistent with the results previously reported [11,23]. Ohashi et al. reported that the plasma adiponectin concentration was positively correlated with an endothelial vasodilation response in mice [15], suggesting that β-conglycinin exerts an antihypertensive affect through an increase in plasma adiponectin concentration. Although the type of dietary protein did not affect the visceral adipose tissue weights in the present study, the gene expression of *Adipoq* in the mesenteric adipose tissue was significantly higher, whereas that of *Pparg* tended to be higher in rats fed the β-CON diet than in those fed the Control diet. Maeda et al. suggested that the *Pparg* ligand was a major regulator for adipocyte function and an increase in adiponectin secretion in adipose tissue [24]. Therefore, the increased *Pparg* expression by feeding of β-conglycinin seemed to lead to an increase in gene expression and synthesis of adiponectin in adipose tissue, resulting in an increased secretion into blood circulation.

In the present study, the plasma NOx concentration tended to be increased by the feeding of β-conglycinin. NO synthesized by endothelial NO synthase (eNOS) has a wide range of biological functions that maintain vascular homeostasis, including modulation of vascular dilator tone and regulation of endothelium-dependent vasorelaxation [25]. Therefore, NO also plays a crucial role in the regulation of blood pressure. The β-conglycinin-dependent increase in plasma NOx concentration in the present study could be due to an increase in plasma adiponectin concentration. In this context, it has been reported that serum/plasma adiponectin contributes to reducing the blood pressure by upregulation of the gene expression of eNOS and by an increase in NO production in vascular endothelial cells [15,26]. Thus, increasing plasma adiponectin concentration by feeding of β-conglycinin was suggested to stimulate the production of eNOS in the aortic endothelium and, consequently, to exert an antihypertensive effect.

Blood pressure is known to be regulated not only by adipocytokines but also by other factors. In the present study, we also investigated the possibility of an antihypertensive effect of β-conglycinin via the renin–angiotensin system. The renin–angiotensin system is known to play a critical role for regulating blood pressure through angiotensin II signaling [27]. Angiotensinogen, the starting substrate of the system, is cleaved by renin to angiotensin I. Angiotensin I is cleaved by ACE to vasoconstrictor angiotensin Ⅱ, which is the end product of the system. However, dietary β-conglycinin did not affect plasma ACE activity in the present study. Yang et al. reported that dietary β-conglycinin significantly decreased the concentration of angiotensin II in plasma and kidney in streptozotocin-induced diabetic nephropathy rats [21]. In the present study, although the gene expressions of *Agt* in the liver and *Ren* in the kidney tended to be higher in the β-CON group than in the Control group, the trends were not significant (*p* = 0.316 for *Agt* and *p* = 0.220 for *Ren*, respectively). Therefore, the involvement of the renin–angiotensin system in the β-conglycinin-dependent antihypertensive effect seemed to be less evident. Ran et al. reported that subcutaneous infusion of angiotensin II for 2 weeks significantly decreased plasma adiponectin concentration, leading to activation of the renin–angiotensin system in rats [28]. It seems likely that the β-conglycinin-dependent increase in plasma adiponectin concentration induces downregulation of the renin–angiotensin system. Therefore, it remains possible that the effect of β-conglycinin is partly modulated by the renin–angiotensin system.

In our previous study in obese Otsuka Long-Evans Tokushima Fatty (OLETF) rats, dietary β-conglycinin decreased visceral adipose tissue weight, decreased triglyceride concentration associated with a decrease in FAS in the liver and increased insulin sensitivity [11]. Consistent with these observations, dietary β-conglycinin decreased triglyceride concentration and cytosolic FAS activity in the liver in the present study. Moreover, dietary β-conglycinin significantly increased the brown adipose tissue weight. Since Das et al. suggested that activation of brown adipose tissue prevents hypertension through secreting hormones, such as fibroblast growth factor 21 [29], the β-CON-dependent increase in brown adipose tissue weight might also contribute to blood pressure suppression. However, the effects on visceral adipose tissue weight and plasma insulin concentration were not clear under the experimental conditions in the present study. The difference in the magnitude of the response to the β-conglycinin-dependent effect could be in part attributed to differences in animal species.

The results in the present study indicated that dietary β-conglycinin suppressed SBP and DBP in the SHR. The effect could in part be attributed to an increase in plasma adiponectin and a concomitant increase in the plasma NO level. Moreover, though the involvement of the β-conglycinin-dependent effect seemed to be less evident, it remains possible that the renin–angiotensin system may participate in the β-conglycinin-dependent antihypertensive effect.

In the present study, rats consumed β-conglycinin at approximately 5.8 g and 1.3 g/day/kg body weight in the β-CON and the SOY groups, respectively. These intakes were estimated to correspond to approximately 0.9 g and 0.2 g/day/kg in humans [30] and were equivalent to 56 g and 13 g/day for a person weighing 60 kg. Based on the consumption of soy and soy products consumed by the Japanese, approximately 60 g/day, the amount of β-conglycinin consumed was calculated to be approximately 4.5 g/day. This amount corresponds to approximately one-third less, even compared with that of the SOY group in the present study. However, since β-conglycinin has already been approved for humans as a triglyceride-lowering food in Japan, at 5 g/day, as an ingredient involved in Food for Specified Health Uses (FOSHU) approved by the Consumer Affairs Agency of the Government of Japan, the use of β-conglycinin may be expected to be a promising food factor for improving hypertension.

## 4. Materials and Methods

### 4.1. Animals and Diets

Five-week-old male spontaneously hypertensive rats (SHR/Izm) were purchased from Japan SLC, Inc. (Shizuoka, Japan). The animals developed spontaneous hypertension at the age of 6 to 19 weeks old [31]. The animals were housed individually in steel cages under controlled conditions (temperature, 22 ± 1 °C.; humidity, 55 ± 5%; light cycle, 08:00–20:00) and fed a commercial chow (MF-2, Oriental Yeast, Co. Ltd., Osaka, Japan). After acclimatizing for 6 days, 18 rats were randomly assigned to 3 groups with 6 animals each, according to the source of dietary protein, casein, soy protein, and β-conglycinin. The diet was prepared according to the AIN-93G formula [32], containing 20% casein (Wako Pure Chemical Industries Ltd., Osaka, Japan) and named the Control diet, as shown in Table 5. For the experimental diet, 50% of the casein in the Control diet was replaced with either soy protein isolate (FUJIPRO, >90% purity, Fuji Oil, Co., Osaka, Japan) or β-conglycinin (LIPOFF-700, >90% purity, Fuji Oil, Co.), and named the SOY diet or the β-CON diet, respectively. The rats were given free access to the diets for 48 days. At the end of the feeding period, the rats fasted for 6 h (03:00–09:00) and were anesthetized with isoflurane and pentobarbital. Blood (9 mL) was collected from the abdominal aorta in a syringe containing 1.0 mL of 3.8% trisodium citrate. The liver, kidney, and adipose tissues were excised, weighed, frozen immediately under liquid nitrogen, and stored at −80 °C until analysis. Plasma was prepared by centrifugation (1000× *g* for 15 min at 4 °C) and stored at −80 °C until used. The experimental procedures were approved by the Animal Use Committee of University of Nagasaki (approval No. 25–24), and the animals were maintained in accordance with the University of Nagasaki guidelines for care and use of laboratory animals.

### 4.2. Measurement of Blood Pressure

During the feeding period, SBP and DBP without preheating were measured every 2 weeks by a tail-cuff method using a blood pressure monitor (Model MK-2000, Muromachi Kikai Co., Tokyo, Japan).

### 4.3. Plasma Analysis

Plasma concentrations of triglyceride, cholesterol, phospholipid, FFA, and glucose were measured with commercial assay kits (Triglyceride E test Wako, Cholesterol E test Wako, Phospholipid C test Wako, NEFA C test Wako, and Glucose CII test Wako, Wako Pure Chemical Industries, Ltd., respectively). Plasma concentrations of adiponectin, insulin, and leptin were measured by enzyme-linked immunosorbent assay using a commercial kit (Mouse/Rat Adiponectin ELISA kit, Otsuka Pharmaceutical, Co. Ltd., Tokyo, Japan; Rat Insulin ELISA kit, Morinaga Institute of Biological Science, Kanagawa, Japan; and Mouse/Rat Leptin ELISA kit, Morinaga Institute of Biological Science, respectively). Plasma concentration of NOx was measured by detecting nitrogen dioxide and nitrogen trioxide, stable metabolites of nitric oxide, using a commercial kit (NO_2_/NO_3_ Assay kit-FX, Dojindo Laboratories, Kumamoto, Japan) after deproteinization of plasma by filtration using an Amicon Ultra Centrifugal Filter (Merck Millipore, Ltd., Carrigtwohill, County Cork, Ireland). Plasma specific activity of angiotensin converting enzyme (ACE) was measured with a commercial kit (ACE color Fujirebio, Inc., Tokyo, Japan). All measurements were performed in accordance with the manufacturer’s instructions. The primary outcomes were adiponectin, NOx, and ACE, and the secondary outcomes were the others.

### 4.4. Liver Lipid Analysis

Total liver lipids were extracted by the method of Folch et al. [33]. Triglyceride and cholesterol concentrations were measured by the method of Carr et al. [34], with minor modifications. Phospholipid concentrations were measured by the method of Rouser et al. [35]. The primary outcome was triglyceride concentration.

### 4.5. Preparation of Hepatic Subcellular Fractions and Measurement of Enzyme Activities

Hepatic subcellular fractions (cytosols, mitochondria/peroxisomes, and microsomes) were prepared by differential centrifugation, as described previously [11]. All subcellular fractions were stored at −80 °C until used. Activities of cytosolic FAS, G6PDH, and malic enzyme in the liver were measured according to the methods reported by Kelley et al. [36], Kelly and Kletzien [37], and Ochoa [38], respectively. The activity of microsomal PAP was measured according to the method reported by Surette et al. [39]. The activity of mitochondrial CPT was also measured according to the methods reported by Bieber et al. [40]. The protein concentrations of the subcellular fractions were measured according to the method reported by Lowry et al. [41]. The primary outcomes were FAS and CPT, and the secondary outcomes were the others.

### 4.6. Total RNA Extraction and Real-Time Polymerase Chain Reaction

Total RNA was isolated from tissues (liver, kidney, and mesenteric adipose tissue) using RNA extraction reagent (Roche Diagnostics GmbH, Mannheim, Germany) and was reverse transcribed into cDNA using Prime Script RT Master Mix (Takara Bio Inc., Shiga, Japan) according to the manufacturer’s instructions. Real-time PCR analysis was performed using the Applied Biosystems 7300 Real-time system (Life Technologies Japan, Ltd., Tokyo, Japan) with SYBR green (Thunderbird^®^ SYBR^®^ qPCR Mix, Toyobo, Co. Ltd., Osaka, Japan). Primer sequences were designed and checked for specificity using the NCBI BLAST [42]. The sequences for the primer pairs used in the present study are listed in Table 6. Target gene expression was measured relative to the expression of acidic ribosomal phosphoprotein (36B4) mRNA (*Rplp0*) as an internal control and determined using the 2^−ΔΔC^*_T_* method [43]. The primary outcomes were *Adipoq* and *Ang*, and the secondary outcomes were the others.

### 4.7. Statistical Analysis

Results are expressed as mean ± SD. All the data were tested for normality using the Kolmogorov–Smirnov test and normality was confirmed (*p* > 0.05). Then, a parametric test was performed using one-way ANOVA followed by the Tukey–Kramer test, and a *p*-value < 0.05 was considered statistically significant (Super ANOVA software, Abacus Concepts, Berkeley, CA, USA).

## 5. Conclusions

The present study indicated that dietary β-conglycinin compared with casein suppressed SBP and DBP through an increase in plasma adiponectin concentration, accompanied by an increase in plasma NOx concentration in the SHR. The involvement of the renin–angiotensin system in the β-conglycinin-dependent antihypertensive effect was less evident. The results provide evidence that dietary β-conglycinin has a beneficial effect on improving hypertension as well as dyslipidemia, even in comparison with soy protein.

## Figures and Tables

**Table 1:** Effect of dietary β-conglycinin on growth parameters in SHR.

	Groups		
	Control	SOY	β-CON
Body weight (g)			
Initial	172 ± 6	171 ± 6	172 ± 9
Final	328 ± 12	328 ± 19	337 ± 14
Food intake (g/day)	19.0 ± 0.9	19.3 ± 0.7	19.7 ± 0.6
Total β-conglycinin consumption (g)	0.0 ± 0.0 *^a^*	20.9 ± 0.8 *^b^*	92.4 ± 3.7 *^c^*
Food efficiency (g body weight gain/g diet)	0.179 ± 0.013	0.177 ± 0.014	0.182 ± 0.006
Tissue weights (g/100 g body weight)			
Liver	3.63 ± 0.16	3.55 ± 0.17	3.71 ± 0.23
White adipose tissue			
Epididymal	1.74 ± 0.14	1.81 ± 0.08	1.80 ± 0.22
Perirenal	1.97 ± 0.28	2.00 ± 0.26	2.00 ± 0.30
Mesenteric	1.21 ± 0.14	1.21 ± 0.18	1.24 ± 0.18
Interscapular brown adipose tissue	0.102 ± 0.010 *^a^*	0.110 ± 0.017 *^a^*^,*b*^	0.123 ± 0.013 *^b^*

Control, 20% casein diet; SOY, the diet containing 10% soy protein isolate with 10% casein; β-CON, the diet containing 10% β-conglycinin with 10% casein. Rats were given free access to the diets for 48 days. Values are expressed as mean ± SD of six rats. *^a,b,c^* Values without sharing a common superscript letter are significantly different at *p* < 0.05.

**Table 2:** Effect of dietary β-conglycinin on plasma concentrations of lipids, glucose, hormones, and factors influencing blood pressure in SHR.

	Groups		
	Control	SOY	β-CON
Triglyceride (mg/dL)	66.7 ± 18.1	54.1 ± 14.7	70.4 ± 24.9
Phospholipid (mg/dL)	117 ± 13	104 ± 9	116 ± 7
Cholesterol (mg/dL)	65.1 ± 4.8 *^a^*	57.2 ± 4.7 *^b^*	59.1 ± 2.9 *^a^*^,*b*^
Free fatty acid (mmol/L)	1.06 ± 0.08 *^a^*	0.940 ± 0.040 *^a^*^,*b*^	0.880 ± 0.069 *^b^*
Glucose (mg/dL)	172 ± 23	158 ± 15	169 ± 10
Insulin (ng/mL)	3.75 ± 1.36	4.03 ± 1.22	2.40 ± 1.33
Adiponectin (μg/mL)	6.06 ± 0.81 *^a^*	6.77 ± 0.50 *^a^*^,*b*^	7.34 ± 0.89 *^b^*
Leptin (ng/mL)	6.98 ± 0.79	6.82 ± 1.12	6.12 ± 0.45
NOx (μmol/mL)	5.15 ± 1.15	5.74 ± 0.88	6.62 ± 1.20
ACE (IU/L)	21.7 ± 2.0	21.5 ± 1.5	20.8 ± 1.5

Control, 20% casein diet; SOY, the diet containing 10% soy protein isolate with 10% casein; β-CON, the diet containing 10% β-conglycinin with 10% casein. Rats were given free access to the diets for 48 days. ACE, angiotensin converting enzyme; NOx, nitric oxide metabolites [NO_2_^−^] + [NO_3_^−^]. Values are expressed as mean ± SD of six rats. *^a^*^,*b*^ Values without sharing a common superscript letter are significantly different at *p* < 0.05.

**Table 3:** Effect of dietary β-conglycinin on liver lipid concentrations in SHR.

	Groups		
	Control	SOY	β-CON
Triglyceride (mg/g liver)	17.6 ± 7.3 *^a^*	10.8 ± 1.2 *^b^*	12.6 ± 2.1 *^a^*^,*b*^
Cholesterol (mg/g liver)	3.33 ± 0.55	3.36 ± 0.37	3.29 ± 0.76
Phospholipid (mg/g liver)	30.0 ± 1.7	28.3 ± 2.5	27.5 ± 2.6

Control, 20% casein diet; SOY, the diet containing 10% soy protein isolate with 10% casein; β-CON, the diet containing 10% β-conglycinin with 10% casein. Rats were given free access to the diets for 48 days. Values are expressed as mean ± SD of six rats. *^a^*^,*b*^ Values without sharing a common superscript letter are significantly different at *p* < 0.05.

**Table 4:** Effect of dietary β-conglycinin on hepatic enzyme activities in SHR.

	Groups		
	Control	SOY	β-CON
	nmol/min/mg protein		
Cytosolic FAS	10.0 ± 0.8 *^a^*	8.17 ± 0.68 *^b^*	8.77 ± 1.05 *^a^*^,*b*^
Cytosolic malic enzyme	31.4 ± 7.9	30.2 ± 3.1	24.7 ± 7.2
Cytosolic G6PDH	45.7 ± 9.5	38.9 ± 4.0	38.6 ± 2.0
Microsomal PAP	4.91 ± 1.23	5.00 ± 0.95	5.43 ± 1.12
Mitochondrial CPT	2.00 ± 0.60	2.50 ± 0.63	2.41 ± 0.50

Control, 20% casein diet; SOY, the diet containing 10% soy protein isolate with 10% casein; β-CON, the diet containing 10% β-conglycinin with 10% casein. Rats were given free access to the diets for 48 days. FAS, fatty acid synthase; G6PDH, glucose-6-phosphate dehydrogenase; PAP, phosphatidate phosphohydrolase; CPT, carnitine palmitoyltransferase. Values are expressed as mean ± SD of six rats. *^a^*^,*b*^ Values without sharing a common superscript letter are significantly different at *p <* 0.05.

**Table 5:** Diet composition (g/kg diet).

	Groups		
	Control	SOY	β-CON
Casein	200	100	100
Soy protein isolate	-	100	-
β-Conglycinin	-	-	100
Cornstarch	200	200	200
Pregelatinized cornstarch	132	132	132
Sucrose	300	300	300
Soybean oil	70	70	70
Cellulose	50	50	50
Mineral mixture (AIN-93G) [32]	35	35	35
Vitamin mixture (AIN-93) [32]	10	10	10
Choline bitartrate	2.5	2.5	2.5
*t*-Butylhydroquinone	0.014	0.014	0.014

**Table 6:** Sequences of gene specific primers used for quantitative real-time PCR.

Names of Genes	Forward Primers (5′ to 3′)	Reverse Primers (5′ to 3′)
*Ang*	CACCTACGTTCACTTCCAAGG	GTGCTGTTGTCCACCCAGAA
*Ren*	TGTAGCTTCAGTCTCCCGACA	GCACTGATCCTGGTCATGTCTAC
*Ace*	ATTGCTTTGGGTGTGGAAGA	GCATCAGAGTAGCCGTTGAG
*Adipoq*	AATCCTGCCCAGTCATGAAG	CATCTCCTGGGTCACCCTTA
*Pparg*	CCCTTTACCACGGTTGATTTCTC	GCAGGCTCTACTTTGATCGCACT
*Rplp0*	GGTGTTTGACAATGGCAGCAT	ATTGCGGACACCCTCTAGGA

*Ang*, angiotensinogen; *Ren*, renin; *Ace*, angiotensin-converting enzyme; *Adipoq*, adiponectin; *Pparg*, peroxisome proliferator-activated receptor γ; *Rplp0*, acidic ribosomal phosphoprotein.

## Data Availability

Data is contained within the article.
